# Carbon dioxide and cardiac output as major contributors to cerebral oxygenation during apnoeic oxygenation

**DOI:** 10.1038/s41598-023-49238-3

**Published:** 2024-02-13

**Authors:** Heiko Andreas Kaiser, Thomas Bauer, Thomas Riva, Robert Greif, Thomas Riedel, Lorenz Theiler, Sabine Nabecker

**Affiliations:** 1 Department of Anaesthesiology and Pain Medicine, Inselspital, Bern University Hospital, University of Bern, Bern, Switzerland; 2 Unit for Research and Innovation, Department of Paediatric Anaesthesia, Istituto Giannina Gaslini, Genova, Italy; 3 University of Bern, Bern, Switzerland; 4 School of Medicine, Sigmund Freud University Vienna, Vienna, Austria; 5 Department of Paediatrics, Cantonal Hospital Graubünden, Chur, Switzerland; 6 Division of Respiratory Medicine, Department of Paediatrics, Inselspital, University Children’s Hospital, University of Bern, Bern, Switzerland; 7 Department of Anaesthesia, Cantonal Hospital Aarau, Aarau, Switzerland; 8 Department of Anaesthesiology and Pain Management, Sinai Health System, University of Toronto, Toronto, Canada

**Keywords:** Outcomes research, Translational research

## Abstract

Apnoeic oxygenation has experienced a resurgence in interest in critical care and perioperative medicine. However, its effect on cerebral oxygenation and factors influencing it, have not yet been investigated in detail. By using near-infrared spectroscopy, we intended to provide further evidence for the safety of apnoeic oxygenation and to increase our understanding of the association between cerebral perfusion, haemodynamic, respiratory and demographic factors. In this secondary analysis of a prospective randomized controlled noninferiority trial, we recruited 125 patients, who underwent surgery under general anaesthesia with neuromuscular blockade. Arterial blood samples were taken every 2 min for a total of 15 min under apnoeic oxygenation with 100% oxygen. Near-infrared spectroscopy and cardiac output were continuously measured. Statistical analysis was performed using uni- and multivariable statistics. Ninety-one complete data sets were analysed. In six patients the SpO_2_ fell below 92% (predefined study termination criterion). The significant average increase of cerebral oxygenation was 0.5%/min and 2.1 mmHg/min for the arterial pressure of carbon dioxide (paCO2). The median cardiac output increased significantly from 5.0 l/min (IQR 4.5–6.0) to 6.5 l/min (IQR 5.7–7.5). The most significant effect on cerebral oxygenation was exhibited by the variable paCO2 and non-specific patient factors, followed by cardiac output and paO2. Apnoeic oxygenation proves to have a high safety profile while significantly increasing cerebral oxygenation, paCO2 and cardiac output. In reverse, NIRS might act as a reliable clinical surrogate of paCO2 and cardiac output during stable arterial oxygenation.

## Introduction

The first investigation of apnoeic oxygenation dates back to Volhard in 1908^1^. In the following years a lot of effort was put into the understanding of apnoeic oxygenation^2–4^, however it failed to show any practical application. With the emergence of high-flow nasal oxygen (HFNO) therapy, apnoeic oxygenation has seen a resurgence in interest as it showed the potential to significantly delay oxygen desaturation for up to 65 min^5,6^ during interventions and procedures without respiration. Although the physiologic mechanism has not yet been completely cleared^5^, this not only reduced the risk of desaturation during difficult airway management or extubation^6^, but also opened up a wide field of interventions and operations, where a tracheal tube would be hindering to the surgeon or interventionalist. The continuous increase of arterial partial pressure of carbon dioxide (paCO2) levels during apnoeic oxygenation might be the most relevant limitation of this technique, as it leads to marked acidosis and increase in cerebral blood flow^7–9^. A new application of HFNO described as Transnasal Humidified Rapid-Insufflation Ventilatory Exchange (THRIVE) reported an end-tidal CO2 increase of only about 1.1 mmHg/min^7^. Recently, in anaesthetized children, there has been evidence, suggesting that apnoeic oxygenation does not significantly increase CO2, whether low flow rates or very high flow rates were used for HFNO^10,11^.

Near-infrared spectroscopy (NIRS) was first introduced in the 1970s as a non-invasive technique to continuously monitor regional tissue oxygenation. This is accomplished by transmission and absorption of light at different wavelengths as it passes through tissue^12–15^. The tissue oxygenation index (TOI) represents the ratio of oxyhaemoglobin to total haemoglobin and is displayed as a percentage value^12,13,16,17^. Numerous studies have been performed to identify factors that influence cerebral oxygenation. Among those are the arterial partial pressure of carbon dioxide (paCO2) and oxygen (paO2)^12,18–20^. The improvement in tissue oxygenation in hypercapnic patients is explained not only with the increase in cerebral blood flow, but also with the accompanying acidosis, which in turn improves oxygen release from haemoglobin (Bohr-effect)^12,15,16^. The haemoglobin concentration itself showed a positive correlation with cerebral oxygenation^21^. Other factors that might influence NIRS values are age and gender. Newman et al. reported in 2020 that all measures of NIRS decline with increasing age and that both oxygenated haemoglobin (O2Hb) and deoxygenated haemoglobin (HHb) measurements are lower in males than females^14^.

In the last couple of years, the NIRS technology has established itself in resuscitation, critical care and during surgery^17,20,22–24^. Especially during cardiac surgery, it has gained substantial supportive evidence^25^. Therefore, numerous studies have tried to find a correlation between cerebral oxygenation and cardiac output (CO)^26–31^. While some claimed a weak correlation^26,28^, others identified a significant influence of CO on cerebral oxygenation^27,29–31^. The purpose of this secondary analysis of data from a randomized controlled noninferiority trial was to provide further evidence for the safety of apnoeic oxygenation and to increase the understanding of the association between cerebral oxygenation and cardiac output, carbon dioxide, age, gender and haemoglobin.

## Methods

### Study cohort

This is a secondary analysis of a randomized controlled noninferiority trial that was approved by the Cantonal Ethics Committee Bern (reference number, ID-2018-00293) and registered with ClinicalTrials.gov (NCT03478774). Written informed consent was obtained from all of the patients before their study enrolment. All research was performed in accordance with relevant guidelines/regulations and was performed in accordance with the Declaration of Helsinki.

Between March 2018 and December 2019 this single-centre study included adult patients with American Society of Anaesthesiologists physical health status I to III who were planned to undergo general anaesthesia for elective surgery at the Department of Anaesthesiology and Pain Medicine, Bern University Hospital, Inselspital, Bern, Switzerland. The individual medical history was gathered during the preoperative anaesthetic assessment. Patients who were considered vulnerable towards hypercapnia and hypoxia due to their comorbidities (e.g., known coronary heart disease, peripheral occlusive arterial disease, anaemia, pregnancy, pulmonary hypertension, increased intracranial pressure, obstructive sleep apnoea, nasal obstruction or hyperkalaemia) were excluded from the study. The primary study was designed to understand a possible ventilatory effect on paCO2 levels during apnoeic oxygenation, its results are presented separately^32^. The detailed inclusion and exclusion criteria can be found in the study protocol^33^ and original publication^32^.

The experiment was terminated once any of the following criteria were met: Arterial oxygen saturation (SpO2) < 92%, transcutaneous pCO2 (tcpCO2) > 100 mmHg, pH < 7.1, potassium (K^+^) > 6.0 mmol/L, or apnoea time reaching 15 min.

### Measurements

On the day of surgery patients were equipped with standardised anaesthesia monitoring, consisting of ECG, pulse oximetry, invasive blood pressure monitoring via peripheral arterial line, end-tidal O2 and end-tidal CO_2_ measurements, transcutaneous pCO2 measurement (TCM, Radiometer, Thalwil, Switzerland), train of four (TOF), electrical impedance tomography (PulmoVista 500; Draeger, Luebeck, Germany) and EEG surveillance (Narcotrend®, Hannover, Germany). To measure cerebral oxygenation we used the forehead near-infrared spectroscopy (NIRS) technique of the NIRO monitor (Niro-200NX, Hamamatsu, Tokyo, Japan), which delivers the tissue oxygenation index (TOI). The TOI was continuously recorded every second. Cardiac output was obtained using pulse contour analysis of the arterial waveform by a LiDCO device (LiDCO, London, UK) with measurements every second. The LiDCO was not calibrated with a Lithium bolus, as the trend of cardiac output was considered important and not the absolute values. Throughout the study, serial arterial blood samples for blood gas analysis (ABL 800, Radiometer, Krefeld, Germany) were drawn and analysed in our central laboratory: awake, immediately after apnoea start and one minute later, then every two minutes during the 15 min apnoeic period.

### Trial design

After arrival in the operating theatre, patients were placed in a supine position, were equipped with the before mentioned monitoring and a peripheral venous cannula was placed. After standard pre-oxygenation (to EtO_2_ > 90%), anesthesia was induced using a target-controlled infusion of propofol and remifentanil. Depth of anesthesia was targeted to Narcotrend index values between 35 and 55. Rocuronium 0.6–0.9 mg/kg was used for neuromuscular blockade; adequacy was verified with a train-of-four value of 0 before onset of apnea and every 5 min throughout the procedure and by visual absence of diaphragmatic movements in the electrical impedance tomography. Hypotension following induction—defined as reduction in mean arterial pressure (MAP) of 20% from preoperative baseline value—was counteracted with a continuous infusion of norepinephrine. After verification that mask ventilation was feasible, a previously sealed opaque envelope containing the randomization was opened and the patients were assigned to one of five groups:

(i) Minimal-flow group: 0.25 L/min oxygen via endotracheal tube (additional study arm, which was added during the study)
(ii) Low-flow group: 2 L/min oxygen + continuous jaw thrust
(iii) Medium-flow group: 10 L/min oxygen + continuous jaw thrust
(iv) High-flow group: 70 L/min oxygen + continuous jaw thrust
(v) Control group: 70 L/min oxygen + continuous laryngoscopy with a McGrath MAC video laryngoscope (Medtronic, Dublin, Ireland).

Inspired oxygen concentration (FiO2) was 1.0 in all patients. In groups ii to iv a senior anaesthesia researcher applied jaw thrust manoeuvre and airway patency was visually confirmed by a nasopharyngeal fiberscope (EF-N slim, Acutronic, Hirzel, Switzerland) three times during the 15 min observation period.

Once assignment to groups has been made and the necessary preparations were completed, mask ventilation was stopped and the start of apnoea was documented as minute 0. Arterial blood samples were taken before and at the start of apnoea, after the first minute and then after every 2 min.

### Statistical analysis

A complete case analysis was performed using the software IBM SPSS Version 27 and R Project for Statistical Computing package (https://www.r-project.org). The obtained data was checked for normality using qq-plots and histograms. Data are presented as median and interquartile range (IQR).

The *outcome variable* of interest in this secondary analysis was the linear change of cerebral oxygenation over the 15 min observational period. The *predictors* investigated on its effect on cerebral oxygenation were patient study-ID (representing non-specific patient factors), group assignment, gender, age, preoperative haemoglobin, linear slopes of paO2, paCO2, cardiac output and MAP. Haemodynamic and NIRS values at the time of the blood samples were averaged over 10 s for further analysis. As a first step univariable analysis with linear regression was used to examine the association of the individual predictors and their trends over the 15 min observational period and cerebral oxygenation. Furthermore, a one-way ANOVA was performed to look into intergroup differences in cerebral oxygenation, paCO2, paO2 and CO, as well as their trends over time.

### Multivariable analysis

Cerebral oxygenation is reported to be significantly influenced by multiple factors that are highly correlated with each other (CO2, mean arterial pressure, cardiac output), so collinearity has to be assumed. Furthermore, due to the study design time is highly correlated with the accumulation of paCO2 as well as inversely correlated with the decline of paO2. Thus, to further examine the effect of these factors on cerebral oxygenation, we performed least absolute shrinkage and selection operator (LASSO) regression to select the most influential predictors. LASSO regression is a technique to reduce model complexity and prevent overfitting. More specifically, we used k-fold cross-validation (k = 10) to optimise the regularisation parameter lambda, which was then implemented to shrink the coefficients for the final model. Thereby, the coefficients of least importance are minimised to zero and removed during the model-finding process^34,35^. The calculations were performed using the glmnet package in R. For this approach we determined the coefficients (slopes) of all continuous numerical predictors and the outcome variable against time by performing simple linear regression. The coefficients were then used to perform the LASSO regression to circumvent the significant effect of time on the above-mentioned variables. To visualize the result of the regression model and the effect of the significantly predictors on cerebral oxygenation, we calculated the mean variable-importance by using 50 permutations and the root-mean-squared-error.

## Results

**Figure 1:**
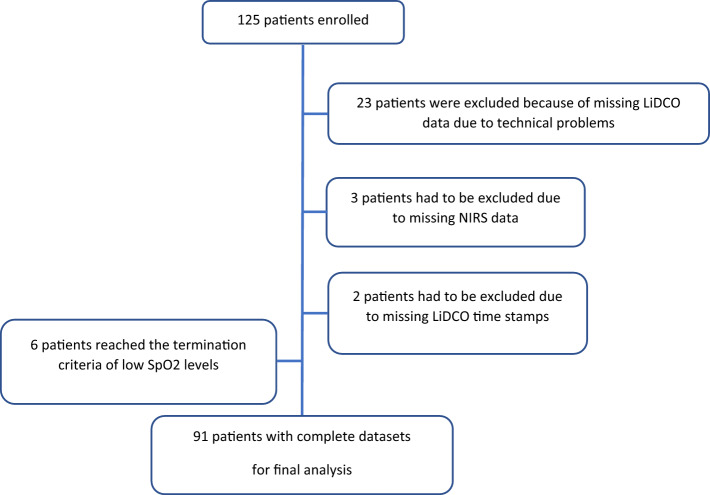
Flow diagram of study population selection.

125 patients were enrolled into the primary study. Six patients (4.7%) reached the termination criteria of deoxygenating below an arterial saturation of 92%. No other termination criteria were met. However, another 28 patients had to be excluded from this secondary analysis due to missing NIRS or LiDCO data. Therefore, a total of 91 patients were included in the statistical analysis (Fig. 1).

### Descriptive statistics

**Figure 2:**
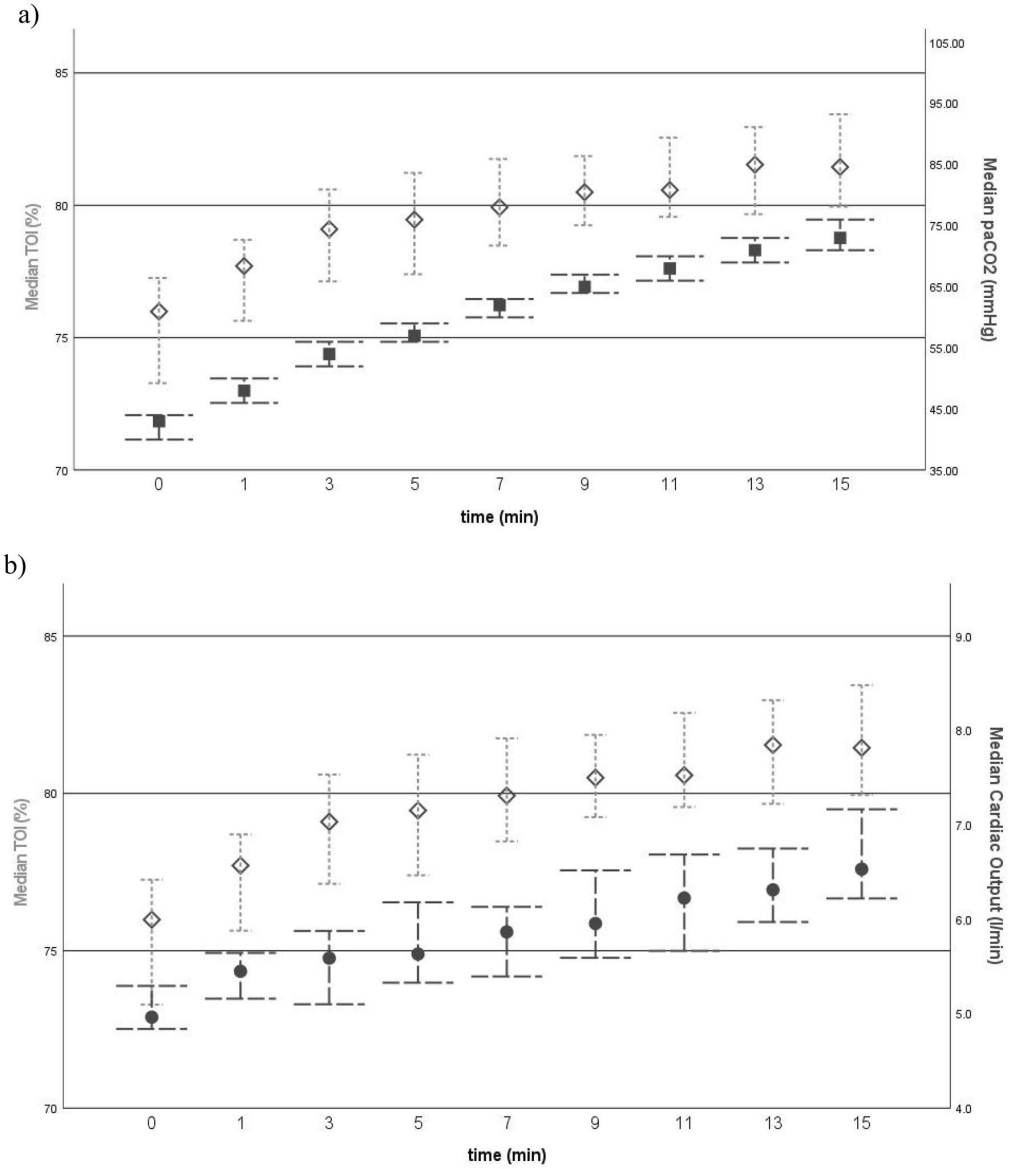
Comparative graphs of (**a**) medians of tissue oxygenation index (TOI) (shown as diamonds) versus arterial partial pressure of carbon dioxide (paCO2) (shown as squares) over time, as well as (**b**) medians of TOI verus cardiac output (CO) (shown as dots) over time; 95% confidence intervals are presented as bars.

The median age of this study population was 47 years (IQR 31 years). Forty-eight were male (52.5%) and 43 female (47.5%). The median TOI at the beginning was 76.0% with an IQR of 9.2%. After 15 min of apnoeic oxygenation median TOI was 81.5% with an IQR of 8.6% (Table 1). The average increase of cerebral oxygenation per minute from baseline was 0.5%/min. Similarly, we observed a steady and significant increase in paCO_2_ from a baseline median of 43 mmHg (IQR 10 mmHg) to 73 mmHg after 15 min (IQR 14 mmHg). The mean change in paCO_2_ from baseline within the 15 min was 2.1 mmHg/min. The baseline median cardiac output was 5.0 l/min (IQR 1.5 l/min) and increased significantly to 6.5 l/min (IQR 1.8 l/min) by minute 15 (Fig. 2). The individual coefficients for each predictor are shown in Table 2. There was no statistical difference in paCO2, TOI or CO increase between the different study groups.

**Table 1:** Descriptive Statistics of predictors.

		Descriptive statistics			Percentiles	
		Median	Minimum	Maximum	25	75
TOI (%)	Min 0	76.0	52.8	86.7	70.3	79.5
	Min 15*	81.5	55.2	96.0	77.7	86.3
paCO2 (mmHg)	Min 0	43	29	59	38	48
	Min 15*	73	55	93	67	81
CO (l/min)	Min 0	5.0	2.3	7.8	4.5	6.0
	Min 15*	6.5	1.9	12.0	5.7	7.5
paO2 (mmHg)	Min 0	366	166	473	323	405
	Min 15	254	78	450	193	320
MAP (mmHg)	Min 0	80	50	133	72	90
	Min 15	80	55	120	71	90
Age		47	18	79	30	61
Hb (g/l)		139	87	179	130	146

*Depicting a significant change from baseline; TOI = tissue oxygenation index; paCO2 = arterial partial pressure of carbon dioxide; paO2 = arterial partial pressure of oxygen; CO = cardiac output; MAP = mean arterial pressure; Hb = haemoglobin.

**Table 2:** Linear regression for each factor on the outcome TOI and the resulting individual coefficients;

Outcome TOI	Coefficient	95% CI	R2	*P*-value
paCO2	0.22	0.18 to 0.25	0.15	*p* < 0.001
paO2	0.004	− 0.00 to 0.01	0.003	*p* = 0.112
CO	0.56	0.26 to 0.87	0.02	*p* < 0.001
MAP	− 0.04	− 0.07 to − 0.01	0.006	*p* = 0.022
Patient	< 0.001	− 0.00 to 0.00	< 0.001	*p* = 0.571
Age	− 0.05	− 0.08 to − 0.03	0.02	*p* < 0.001
Group				
2	–		0.09	*p* < 0.001
3	− 0.01	− 1.39 to 1.38		*p* = 0.991
4	− 4.16	− 5.59 to − 2.73		*p* < 0.001
5	− 1.44	− 3.12 to 0.23		*p* = 0.092
1	− 4.71	− 6.14 to − 3.28		*p* < 0.001
Gender	− 2.93	− 3.86 to − 2.00	0.04	*p* < 0.001
Hb	0.08	0.05 to 0.12	0.03	*p* < 0.001

95% CI = 95% confidence interval; TOI = tissue oxygenation index; paCO2 = arterial partial pressure of carbon dioxide; paO2 = arterial partial pressure of oxygen; CO = cardiac output; MAP = mean arterial pressure; Hb = haemoglobin.

### Multivariable analysis

**Figure 3:**
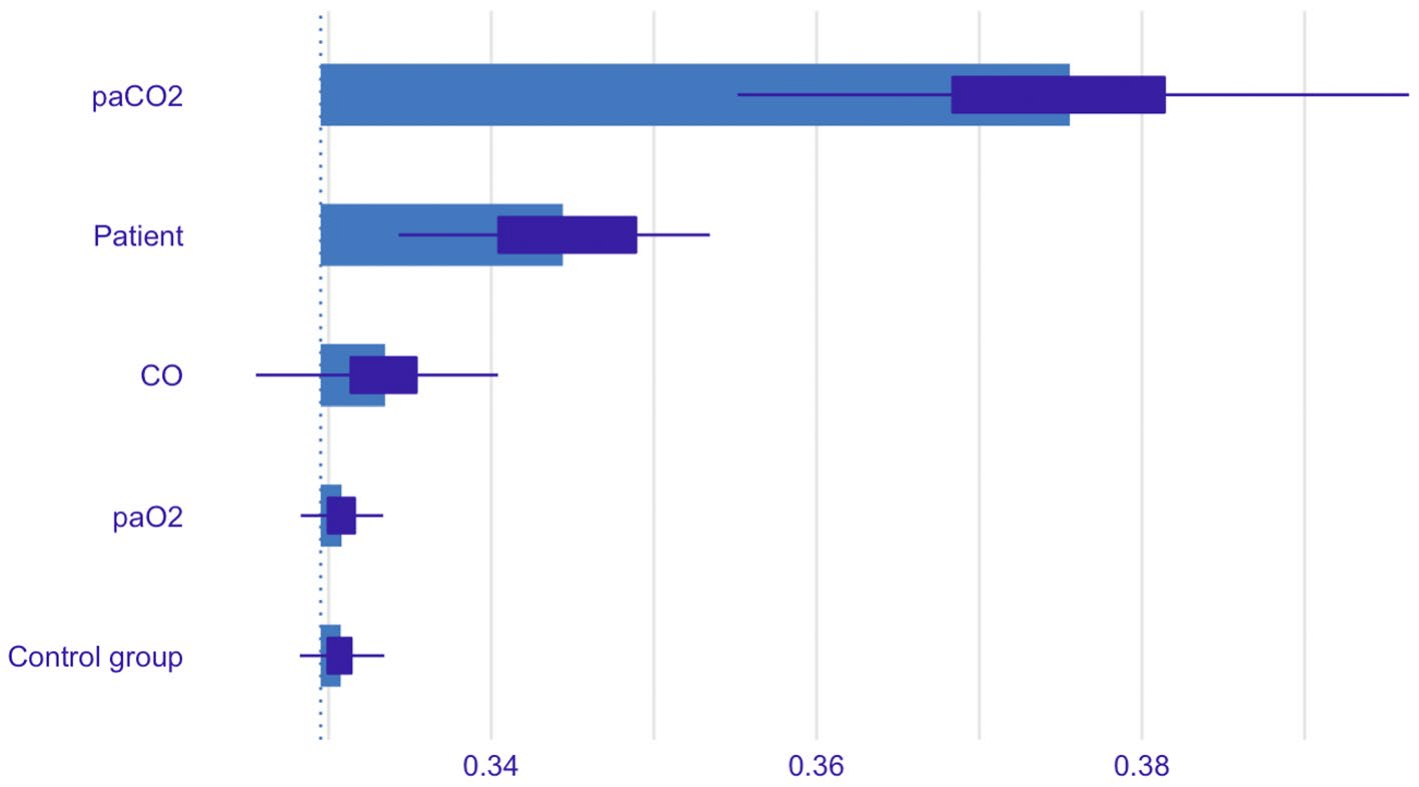
Mean variable importance calculated by using 50 permutations and the root-mean-squared-error loss-function for the LASSO regression model for the effect on cerebral oxygenation. The bars in the plot indicate the mean values of the variable-importance measures for the explanatory variables. Box plots are added to the bars to provide an idea about the distribution of the values of the measure across the permutations.

The LASSO regression revealed that non-specific patient factors, the arterial partial pressures of carbon dioxide and oxygen, as well as cardiac output and group assignment explain 65% of the variation and kinetic of cerebral oxygenation (R2 = 0.646) in this study. Concerning group assignment, the control group with high-flow oxygen at 70 L/min via Optiflow with continuous laryngoscopy had a significant influence on cerebral oxygenation, while the other groups did not. As presented in the variable importance analysis (Fig. 3), the main effect seems to be mediated by paCO2 and non-specific patient factors—represented as patient study-ID—and less by cardiac output. The arterial partial pressure of oxygen—as long as the arterial oxygen saturation levels are above 92%—and the control group are associated with minor but significant effects on cerebral oxygenation.

In contrast, patient age, gender, haemoglobin level and blood pressure were not associated with a significant change of cerebral oxygenation.

## Discussion

In this secondary analysis of a randomized controlled noninferiority trial, we observed a steady and significant increase in cerebral oxygenation of 0.5%/min during 15 min of apnoeic oxygenation. The univariable and multivariable analysis indicate a major contribution of the increasing paCO2 and cardiac output (Figs. 2 and 3) to this kinetic. While paCO2 has consistently been reported as having a major influence on cerebral oxygenation and cerebral blood flow (CBF)^36^, CO failed repeatedly to prove so due to methodological issues. Many studies used alterations of the central blood volume to acutely and significantly decrease CO or by using magnetic resonance imaging techniques in a resting state^37^. While some studies showed a significant correlation between hemodynamic parameters and cerebral tissue oxygenation^27,29–31^, others did not^26,28^. In this regard our study could verify that NIRS is a clinically valuable mirror of CO in the end. At physiological levels of paCO2, CO might even present a more significant effect on cerebral oxygenation.

Interestingly, the multivariable analysis revealed that the control group (O2 flow of 70 l/min with continuous laryngoscopy) had a significant effect on cerebral oxygenation. The only difference was the use of continuous laryngoscopy in that group, whereas in all other groups airway patency was achieved by jaw thrust. We might speculate that this difference was introduced by the continuous painful stimulus of laryngoscopy—despite intended sufficient analgesia—possibly leading to higher catecholamine levels and cardiac output.

Another major influencing factor of cerebral oxygenation is the patient itself. There are individual differences described in distance from skin surface to the brain, skin perfusion, as well as anatomical differences in position and size of the frontal sinus. Also, slight differences in the positioning of the probes between individuals might have been significant contributors to detected the changes in cerebral oxygenation^38–40^.

Neither of the other measured parameters showed any relevant effect on tissue oxygenation on the multivariable level. In accordance to previous studies, we observed a positive correlation between Hb and cerebral oxygenation in the univariable analysis. The question remains, how much a sudden decrease in haemoglobin due to intraoperative bleeding would affect cerebral oxygenation, as oxygen delivery is essentially depending on it. To answer this, intraoperative NIRS data with continuous Hb measurement should be looked at in future studies.

Especially during cardiac surgery, it is known that mean arterial pressure is correlated with cerebral oxygenation as soon as the pressure is lower or higher than the lower or upper limit of cerebral autoregulation^39^. As we kept the MAP within normal limits it is not surprising that MAP was not a significant contributor to cerebral oxygenation in this study. With blood pressure being within the range of autoregulation, no severe changes on cerebral perfusion were to be expected. Further studies are necessary to evaluate the safety of apnoeic oxygenation in patients with cerebral pathologies and unstable haemodynamics.

As arterial saturation was within physiological limits during the trial, no major but still significant contribution of paO2 to cerebral oxygenation was observed.

The group assignment was not an essential part of the design for this secondary analysis. Carbon dioxide clearance under different oxygen flow rates was the primary endpoint of the randomized controlled noninferiority trial published separately^32^. The expected increase of cerebral oxygenation during apnoeic oxygenation and its influencing factors were the central point of interest of the discussed investigation herein. Thus, we did not present the results split into different groups, but as the group assignment was part of the original study design, we still had to adjust for it in the regression analysis.

## Limitations

This analysis included a rather healthy surgical patient population, excluding anyone with pre-existing pulmonary conditions. This and the fact that only elective cases were included, limits the transfer of results to patients with known lung diseases or other major health issues. Obviously, the anaesthesiologist in charge was not blinded, however this should not have had any effect on the outcome of cerebral oxygenation.

## Conclusion

Cerebral oxygenation increases significantly during apnoeic oxygenation in association with rising arterial CO_2_ levels. Concomitantly, we found important clinical evidence of a significant impact of cardiac output on cerebral NIRS as a surrogate parameter of cerebral oxygenation and perfusion. The increasing cardiac output and cerebral oxygenation during apnoeic oxygenation of 15 min are a solid indicator of safety for this practice.

## Data Availability

The datasets used and/or analysed during the current study are available from the corresponding author on reasonable request after Ethics committee approval.

## References

[1] Volhard F (1908). Über künstliche Atmung durch Ventilation der Trachea und eine einfache Vorrichtung zur rhythmischen künstlichen Atmung. Münchener Medizinische Wochenschrift.

[2] Fraioli RL, Sheffer LA, Steffenson JL (1973). Pulmonary and cardiovascular effects of apneic oxygenation in man. Anesthesiology.

[3] Kolettas A, Grosomanidis V, Kolettas V (2014). Influence of apnoeic oxygenation in respiratory and circulatory system under general anaesthesia. J. Thorac. Dis..

[4] Roth LW, Whitehead RW, Draper WB (1947). Studies on diffusion respiration; Survival of the dog following a prolonged period of respiratory arrest. Anesthesiology.

[5] Riva T, Meyer J, Theiler L (2021). Measurement of airway pressure during high-flow nasal therapy in apnoeic oxygenation: A randomised controlled crossover trial*. Anaesthesia.

[6] Ricard J-D, Roca O, Lemiale V (2020). Use of nasal high flow oxygen during acute respiratory failure. Intens. Care Med..

[7] Patel A, Nouraei SAR (2015). Transnasal Humidified Rapid-Insufflation Ventilatory Exchange (THRIVE): A physiological method of increasing apnoea time in patients with difficult airways. Anaesthesia.

[8] Gustafsson IM, Lodenius Ã, Tunelli J (2017). Apnoeic oxygenation in adults under general anaesthesia using Transnasal Humidified Rapid-Insufflation Ventilatory Exchange (THRIVE)—A physiological study. Br. J. Anaesth..

[9] Bain AR, Ainslie PN, Hoiland RL (2016). Cerebral oxidative metabolism is decreased with extreme apnoea in humans; Impact of hypercapnia. J. Physiol..

[10] Riva T, Pedersen TH, Seiler S (2018). Transnasal humidified rapid insufflation ventilatory exchange for oxygenation of children during apnoea: A prospective randomised controlled trial. Brit. J. Anaesth..

[11] Riva T, Préel N, Theiler L (2020). Evaluating the ventilatory effect of transnasal humidified rapid insufflation ventilatory exchange in apnoeic small children with two different oxygen flow rates: A randomised controlled trial*. Anaesthesia.

[12] Marin T, Moore J (2011). Understanding near-infrared spectroscopy. Adv. Neonatal Care.

[13] Murkin JM, Arango M (2009). Near-infrared spectroscopy as an index of brain and tissue oxygenation. Br. J. Anaesth..

[14] Newman L, Nolan H, Carey D (2020). Age and sex differences in frontal lobe cerebral oxygenation in older adults—Normative values using novel, scalable technology: Findings from the Irish Longitudinal Study on Ageing (TILDA). Arch. Gerontol. Geriat..

[15] Suemori T, Skowno J, Horton S (2016). Cerebral oxygen saturation and tissue hemoglobin concentration as predictive markers of early postoperative outcomes after pediatric cardiac surgery. Pediatr. Anesth..

[16] Wong FY, Alexiou T, Samarasinghe T (2010). Cerebral arterial and venous contributions to tissue oxygenation index measured using spatially resolved spectroscopy in newborn lambs. Anesthesiology.

[17] Nagdyman N, Fleck T, Barth S (2004). Relation of cerebral tissue oxygenation index to central venous oxygen saturation in children. Intens. Care Med..

[18] Tisdall MM, Taylor C, Tachtsidis I (2009). The effect on cerebral tissue oxygenation index of changes in the concentrations of inspired oxygen and end-tidal carbon dioxide in healthy adult volunteers. Anesth. Analg..

[19] Jakkula P, Reinikainen M, Group C Study (2018). Targeting two different levels of both arterial carbon dioxide and arterial oxygen after cardiac arrest and resuscitation: A randomised pilot trial. Intens. Care Med..

[20] Sandroni C, Parnia S, Nolan JP (2019). Cerebral oximetry in cardiac arrest: A potential role but with limitations. Intens. Care Med..

[21] Kishi K, Kawaguchi M, Yoshitani K (2003). Influence of patient variables and sensor location on regional cerebral oxygen saturation measured by INVOS 4100 near-infrared spectrophotometers. J. Neurosurg. Anesth..

[22] Bhatia A, Gupta AK (2007). Neuromonitoring in the intensive care unit. II. Cerebral oxygenation monitoring and microdialysis. Intens. Care Med..

[23] Ghanayem NS, Hoffman GM (2016). Near infrared spectroscopy as a hemodynamic monitor in critical illness. Pediatr. Crit. Care Med..

[24] Bösel J, Purrucker JC, Nowak F (2012). Volatile isoflurane sedation in cerebrovascular intensive care patients using AnaConDa®: effects on cerebral oxygenation, circulation, and pressure. Intens. Care Med..

[25] Menke J, Möller G (2014). Cerebral near-infrared spectroscopy correlates to vital parameters during cardiopulmonary bypass surgery in children. Pediatr. Cardiol..

[26] Dullenkopf A, Baulig W, Weiss M, Schmid ER (2007). Cerebral near-infrared spectroscopy in adult patients after cardiac surgery is not useful for monitoring absolute values but may reflect trends in venous oxygenation under clinical conditions. J. Cardiothor. Vasc. An..

[27] Vretzakis G, Georgopoulou S, Stamoulis K (2014). Cerebral oximetry in cardiac anesthesia. J. Thorac. Dis..

[28] Al-Subu AM, Hornik CP, Cheifetz IM (2018). Correlation between regional cerebral saturation and invasive cardiac index monitoring after heart transplantation surgery. J. Pediatr. Intens. Care.

[29] Desmond F, Namachivayam S (2016). Does near-infrared spectroscopy play a role in paediatric intensive care?. Bja Educ..

[30] Jo YY, Shim J-K, Soh S (2020). Association between cerebral oxygen saturation with outcome in cardiac surgery: Brain as an index organ. J. Clin. Med..

[31] Paquet C, Deschamps A, Denault AY (2008). Baseline regional cerebral oxygen saturation correlates with left ventricular systolic and diastolic function. J. Cardiothor. Vasc. An..

[32] Riva T, Robert G, Heiko K (2021). Carbon dioxide clearance during high-flow nasal oxygenation in apneic patients: A single-center randomized controlled noninferiority trial. Anesthesiology.

[33] Theiler L, Schneeberg F, Riedel T, Kaiser H, Riva T, Greif R (2019). Apnoeic oxygenation with nasal cannula oxy- gen at different flow rates in anaesthetised patients: A study protocol for a non-inferiority randomised controlled trial. BMJ Open.

[34] Steyerberg EW, Harrell FE (2016). Prediction models need appropriate internal, internal-external, and external validation. J Clin. Epidemiol..

[35] Hastie TJ, Tibshirani RJ, Friedman JJH (2009). The Elements of Statistical Learning.

[36] Kety SS, Schmidt CF (1948). The effects of altered arterial tensions of carbon dioxide and oxygen on cerebral blood flow and cerebral oxygen consumption of normal young men 1. J. Clin. Invest..

[37] Meng L, Hou W, Chui J (2015). Cardiac output and cerebral blood flow: The integrated regulation of brain perfusion in adult humans. Anesthesiology.

[38] Okada E, Delpy DT (2003). Near-infrared light propagation in an adult head model. II. Effect of superficial tissue thickness on the sensitivity of the near-infrared spectroscopy signal. Appl. Opt..

[39] Vranken NPA, Weerwind PW, Sutedja NA (2017). Cerebral oximetry and autoregulation during cardiopulmonary bypass: A review. J. Extra-corpor. Technol..

[40] Rummel C, Basciani R, Nirkko A (2018). Spatially extended versus frontal cerebral near-infrared spectroscopy during cardiac surgery: A case series identifying potential advantages. J. Biomed. Opt..

